# Reshaping of Bilateral Gait Coordination in Hemiparetic Stroke Patients After Early Robotic Intervention

**DOI:** 10.3389/fnins.2018.00719

**Published:** 2018-10-09

**Authors:** Sandra Puentes, Hideki Kadone, Hiroki Watanabe, Tomoyuki Ueno, Masashi Yamazaki, Yoshiyuki Sankai, Aiki Marushima, Kenji Suzuki

**Affiliations:** ^1^Faculty of Engineering, Information and Systems, University of Tsukuba, Tsukuba, Japan; ^2^Center for Innovative Medicine and Engineering, University of Tsukuba Hospital, Tsukuba, Japan; ^3^Center for Cybernics Research, University of Tsukuba, Tsukuba, Japan; ^4^Department of Rehabilitation Medicine, University of Tsukuba Hospital, Tsukuba, Japan; ^5^Department of Orthopaedic Surgery, University of Tsukuba Hospital, Tsukuba, Japan; ^6^Department of Neurosurgery, University of Tsukuba Hospital, Tsukuba, Japan

**Keywords:** stroke, hemiparesis, robot suit HAL, gait coordination, early intervention

## Abstract

Hemiparetic gait is a common condition after stroke which alters importantly the quality of life of stroke survivors. In recent years, several robotic interventions have been developed to support and enhance rehabilitation strategies for such population. The Hybrid Assistive Limb^®^ (HAL) robot suit is a unique device able to collect in real time bioelectric signals from the patient to support and enhance voluntary gait. HAL has been used before in early stages of stroke showing gait improvement after the intervention. However, evaluation of the coordination of gait has not been done yet. Coordination is a key factor for an adequate gait performance; consequently, its changes may be closely related to gait recovery. In this study, we used planar covariation to evaluate coordination changes in hemiparetic stroke patients after early HAL intervention. Before starting, impaired intersegmental coordination for the paretic and non-paretic side was evident. HAL intervention was able to induce recovery of the covariation loop shape and deviation from the covariation plane improving intersegmental coordination. Also, there was a tendency of recovery for movement range evidenced by comparison of peak elevation angles of each limb segment of the patients before and after HAL intervention, and also when compared to healthy volunteers. Our results suggest that early HAL intervention contributed to the improvement of gait coordination in hemiparetic stroke patients by reinforcing central pattern generators and therefore reshaping their gait pattern.

**Trial registration:** UMIN000022410 2016/05/23.

## Introduction

Stroke is known as a leading cause of motor impairment, whose burden in terms of disability continue increasing worldwide (Feigin et al., 2017; Benjamin et al., 2018). Exercise is the most common intervention to improve walking performance (Eng and Tang, 2007) and previous studies have shown proof that early interventions designed to be intense and repetitive depending on the patient tolerance are able to improve functional outcome when compared to conventional rehabilitation (Salter et al., 2006; Eng and Tang, 2007; Peurala et al., 2007). Additionally, it has been found that patients admitted within the first 30 days of stroke onset to rehabilitation programs experience greater functional improvement accompanied by reduction in length of hospital stay (Salter et al., 2006). In recent years, the development of new technologies to improve the outcome of exercise training in patients with gait disturbances has opened the path to the development of variated exoskeleton models (Chen et al., 2013; Esquenazi et al., 2017).

The Hybrid Assistive Limb^®^ (HAL) robot suit, is a wearable exoskeleton able to provide bilateral or unilateral leg support. The single-leg version is used to assist patients presenting hemiparesis. This HAL version has three degrees of freedom related to the sagittal movement of hip, knee, and ankle joints of the braced side. Using surface electrodes, HAL is able to collect bioelectric signals from the action potentials reaching flexor and extensor muscles of the hip and knee during movement preparation and initiation. This information is processed by an on-board processor to control two motors located over the lateral aspects of the supported hip and knee joints, respectively, in order to assist the voluntary control of the patient joints motion during gait training in real time (Kawamoto et al., 2013). Previous studies have established the feasibility and safety of HAL intervention in early stages of stroke (Ueba et al., 2013; Ogata et al., 2015). These studies did not report major secondary effects. However, patients with intracerebral hemorrhage and low functional scores tended to present orthostatic hypotension. Regarding performance and HAL intervention, one study examining intracerebral hemorrhage patients found better outcome for patients with right intracerebral hemorrhage exclusively when compared to patients who received standard rehabilitation (Ogata et al., 2015); however, the authors reported that HAL intervention group displayed larger hematoma volumes and a trend of increased severity at the initial evaluation when compared to control group (Ogata et al., 2015). Other study including ischemic and hemorrhagic stroke patients reported gait improvement, better torso posture and ability to stand with HAL assist for patients treated with the exoskeleton (Ueba et al., 2013).

There are previous studies using other robotic interventions implemented within the first 30 days after stroke onset. Patients with severe impairment of the motor function using the end-effector-type robotic device denominated as Gait Trainer showed improvement of the motor outcome which was sustained after 2 years (Peurala et al., 2007; Morone et al., 2012). The exoskeleton Lokomat was able to induce motor recovery and improvement of cardiopulmonary fitness which is an issue for bedridden patients (Chang et al., 2012). Finally, a study implementing the gait assistance robot GAR found motor and functional recovery but it was not significantly different from regular rehabilitation; however, the extensor muscle torque improved bilaterally, being significant only for the non-paretic side (Ochi et al., 2015).

These studies focused their analysis on the comparison of functional scales and gait parameters, but there are no additional measurements to evaluate coordination changes after robotic intervention. It is known that coordination impairment is an underlying cause of gait deficit after stroke (Bleyenheuft et al., 2009; Chow and Stokic, 2015); hemiparesis alters the stabilization of head and thorax which contribute to the deviation of walking trajectories and poor balance during gait generation (Lamontagne et al., 2005). Also, muscle weakness from the hemiparetic side affect the initiation of movement and proper flexion and extension of the ipsilateral hip, knee, and ankle (Quervain et al., 1996). Likewise, post-stroke patients have more difficulties regulating their walking speed, step frequency, and step length which are important elements to execute stable gait when walking in complex environments (Hak et al., 2013). However, despite unilateral brain damage, abnormal patterns of movement are also found in the non-paretic side during gait generation (Quervain et al., 1996) accompanied by increment of the bilateral kinematic variability. These changes have been associated to asymmetry and lack of coordination of the left-right stepping phase (Meijer et al., 2011). Additionally, these conditions impair the ability to avoid obstacles bilaterally, becoming more prominent under time pressure (Otter et al., 2005). Bilateral coordination is an important component of gait pattern; therefore, evaluation of its changes may help to elucidate the impact of robotic interventions.

Kinematic analysis of gait has been attempted before by measuring changes in joint angles; however, the pattern of the flexion-extension angles of hip and knee joints tends to generate a large variation in inter-patients and inter-trial results (Boudarham et al., 2013) and becomes dependent on the gait speed (Borghese et al., 1996). Since the gait pattern is drastically altered after stroke due to hemiparesis and march instability, perturbing patients’ balance and speed (Quervain et al., 1996; Goldie et al., 2001); it is ideal to collect data using a method able to obtain reproducible results despite these gait characteristics. Analysis of the EA of the lower limbs has shown a stereotyped pattern in healthy volunteers despite gait pattern, speed variation, or anatomic discrepancies (Borghese et al., 1996; Ivanenko et al., 2008). The EA are calculated by the relationship of lower limb thigh, shank, and foot segments to the vertical. When plotted against each other, these angles covary describing regular loops over a plane (Borghese et al., 1996; Ivanenko et al., 2008). The correlation of the coordination patterns among the EA of the lower limb segments during locomotion is known as the law of intersegmental coordination (Borghese et al., 1996; Barliya et al., 2009). It has been suggested that this planar law represents the coordinated kinematic synergies of the body in charge of the maintenance of dynamic equilibrium during gait progression and anticipatory locomotor adjustments to environmental changes (Maclellan and McFadyen, 2010). This analysis has been previously used for gait analysis of stroke patients (Bleyenheuft et al., 2009; Chow and Stokic, 2015) and we previously reported a planar covariation analysis of myelopathy patients after HAL intervention (Puentes et al., 2018); however; to our knowledge, this is the first study examining planar covariation after an early robotic intervention as a measure of gait coordination in stroke patients.

## Materials and Methods

### Participants

Eleven patients hospitalized with a diagnosis of acute stroke were selected for this study (8 women and 3 men). As inclusion criteria, patients within 40 to 80 years old, displaying hemorrhagic or ischemic stroke causing hemiparesis; with a stroke onset within 7 to 14 days were selected. Only first-time episode patients with a FAC higher than 4 before the stroke onset, able to give consent for the study and physically suitable for HAL fitting were included. All patients were able to control their lower extremities voluntarily, but minimal weight support was provided if necessary. One patient was excluded due to anxiety over using an exoskeleton and interference with a previous spinal surgery (included patients *n* = 10, age 60.7 ± 11 years; **Table 1**). As control, kinematics data from nine healthy age-matched volunteers (5 women and 4 men, age 58.2 ± 11 years; **Table 1**) was used. This study was approved by the Ethics Committee of the University of Tsukuba Hospital (approval number: H27-257) and implemented according to the ethical principles stipulated in the Declaration of Helsinki and the University Guidelines for Clinical Trials. All patients received a personalized explanation of the study, participation and data usage before signing an informed consent.

**Table 1:** Characteristics of the participants.

ID	Included participants	Gender	Age	Diagnostic	Lesion	Paretic side	Interval (days)
S1	Stroke	F	67	Atherothrombotic cerebral infarction	Posterior limb of the internal capsule	Left	10
S2	Stroke	F	52	Intracerebral hemorrhage (subcortical)	Parietal lobe	Right	17
S3	Stroke	F	71	Brain stem infarction	Pons: paramedial left side	Left	11
S4	Stroke	M	55	Lacunar infarct	Right basal ganglia to corona radiata	Left	10
S5	Stroke	F	55	Atherothrombotic cerebral infarction	Anterior cerebral artery territory	Left	16
S6	Stroke	M	43	Lacunar infarct	Lateral thalamus	Right	11
S7	Stroke	F	51	Atherothrombotic cerebral infarction	Left basal ganglia to corona radiata	Right	18
S8	Stroke	M	80	Atherothrombotic cerebral infarction	Putamen	Right	16
S9	Stroke	F	61	Cerebral hemorrhage	Thalamus, third ventricle	Left	12
S10	Stroke	F	72	Hypertensive intracerebral hemorrhage	Thalamus	Right	14
H1	Healthy	F	56	–	–	–	–
H2	Healthy	F	42	–	–	–	–
H3	Healthy	F	59	–	–	–	–
H4	Healthy	F	67	–	–	–	–
H5	Healthy	F	60	–	–	–	–
H6	Healthy	M	50	–	–	–	–
H7	Healthy	M	45	–	–	–	–
H8	Healthy	M	77	–	–	–	–
H9	Healthy	M	68	–	–	–	–

*Interval refers to the days elapsed from stroke onset to the beginning of HAL intervention. One patient excluded from the study is not listed.*

### HAL Intervention

The intervention consisted in 9 HAL sessions performed within the hospitalization period three times per week. Each session started with HAL fitting and both patient and robot were secured to the All-In-One^®^ walking trainer (Ropox A/S, Naestved, Denmark). This support system provides individual harnesses for the patient and the robot in order to ensure safety during the sessions and support the robot weight (14 kg). Once the patient was ready, the HAL treatment started consisting of 20 min of walking activity in a 25 m oval course at a comfortable pace. The condition of each patient was assessed by monitoring their blood pressure, heart rate, and oxygen saturation at the beginning, end, and within treatment intervals to ensure that patients were stable. At the end of the treatment, the patients were released from the robot and harness. In total, HAL intervention takes about 1 h from HAL fitting to release.

### HAL Configuration

The single leg version of the robot suit HAL was used to support the hemiparetic side of stroke patients only. The neuromuscular activity of the Iliopsoas (hip flexor), Gluteus Maximus (hip extensor), Biceps Femoris (knee flexor), and the Quadriceps (vastus lateralis and knee extensor) of the affected side was detected through surface electrodes and processed by HAL to assist the patients’ gait. The robot locates two motors beside the patient’s hip and knee; when actuated by muscle signals, these motors are able to produce torque in proportion to the weighted difference of filtered activation of the ipsilateral flexor and extensor muscles corresponding to the hip and knee, respectively. The weights multiplied on the activation of the antagonistic muscles and the overall gain were adjusted individually depending on patient’s electromyography signals, gait performance, and verbally reported subjective perception through HAL intervention sessions.

### Evaluation

**FIGURE 1:**
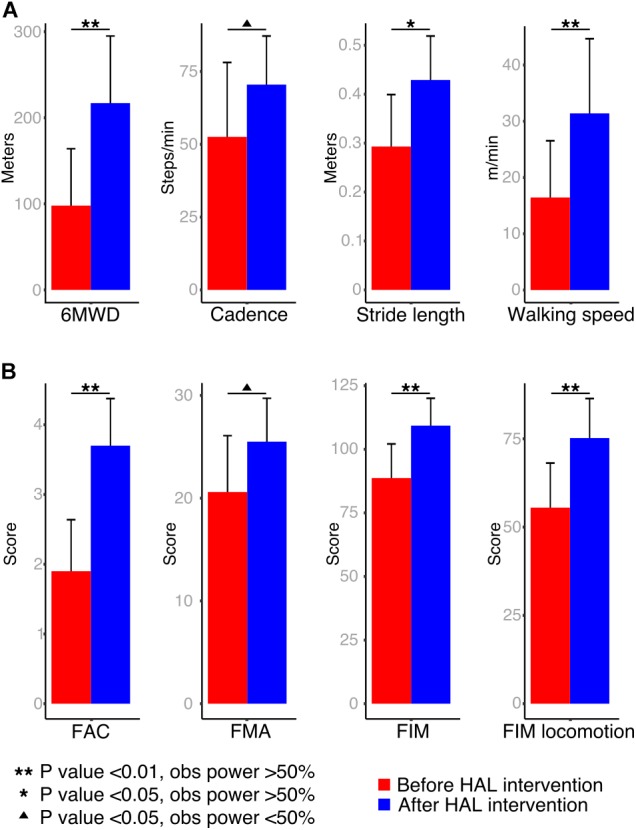
Walking performance and clinical scores: **(A)** patients were assessed during a 6-min walking distance test and cadence, stride length and walking speed were calculated from recordings. **(B)** To evaluate the degree of functionality of patients, the Functional Ambulation Classification (FAC), Fugl-Meyer Assessment (FMA), Functional Independence Measure (FIM) total, and locomotion scores were performed.

Before starting the first HAL session and after the last one, the degree of dependence on daily life activities of stroke patients was evaluated by using the FAC, FMA, and the FIM (total and locomotion only). Following, the patients were evaluated by using the 6MWD. Recordings were used to calculate the stride length, cadence, and speed (**Figure 1**). All evaluations were performed without using HAL.

### Data Collection and Analysis

**FIGURE 2:**
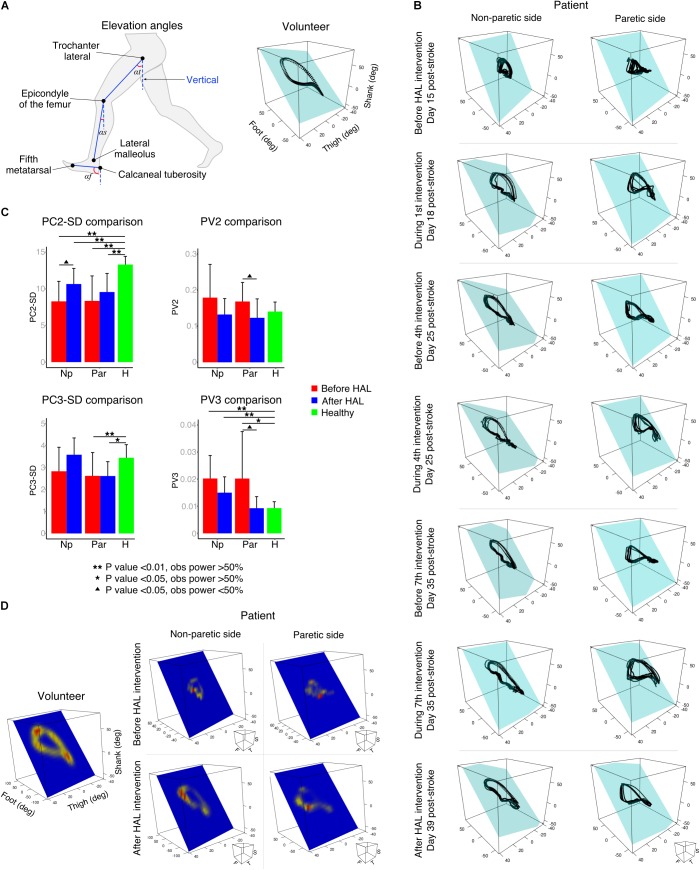
Planar covariation of elevation angles. **(A)** Left: thigh, shank, and foot limb segments used to calculate the elevation angles. Right: planar covariation sample from one healthy volunteer leg. **(B)** Planar covariation sample from one patient paretic and non-paretic leg before, during and after HAL intervention. Each dotted line corresponds to different strides of the same limb **(C)** PCA comparisons using second (PC2) and third (PC3) components. Comparisons of the actual data were done with the standard deviation (SD). The proportional variance of both components was also analyzed (PV2 and PV3). **(D)** Heat maps were used to plot the deviation from the plane for each participant. Sample from one volunteer leg and one patient paretic and non-paretic leg before and after HAL are shown.

Segmental kinematics were recorded by using a motion capture system (VICON MX, 16 T20s cameras, 100 Hz) with Plug-in Gait lower limbs marker-set before the first and after the last HAL intervention for 10 stroke patients, and during a single session for 9 healthy volunteers. For data collection, patients were asked to walk 10 m over a straight line; the initial and final steps were discarded to collect the steps with best performance. Six to 24 cycles were used for analysis. For the last three patients, segmental kinematics were recorded on days 1, 4, and 7 before and during HAL intervention. Since the number of patients was not enough for statistical comparison, only data from one patient was used as an example of planar covariation changes before, during, and after HAL intervention. The EA of the lower limbs were analyzed regarding the orientation of the limb segments in the sagittal plane with respect to the vertical as described before (Borghese et al., 1996). The evaluated segments (**Figure 2A**) were denominated as thigh (from the trochanter to the lateral epicondyle of the femur), shank (from the lateral epicondyle of the femur to the lateral malleolus), and foot (from the posterior calcaneal tuberosity to the second metatarsal). Following, planar covariation of the EA for each lower limb was calculated by using a principal component (PC) analysis after data normalization by subtracting the mean value.

Previous studies have described in healthy volunteers that the first (PC1) and second (PC2) components covariates over a plane describing the shape of the gait loop; nevertheless, the third component (PC3) is orthogonal to the plane, representing the data component that deviates from the covariance plane (Borghese et al., 1996). We calculated the standard deviation of PC2 and PC3 (PC2-SD and PC3-SD) to evaluate the actual width of the covariance loop and the deviation from the covariance plane, respectively. Additionally, we evaluated the proportional width of the covariance loop determined by the percentage of variance (PV) of the PC2 (PV2) and the proportional deviation from the plane determined by the PV of PC3 (PV3) as described before (Martino et al., 2014). The PV3 becomes an index of planarity of the loop, where 0% corresponds to an ideal plane fitting, thus evaluating the proportional deviation from the covariance plane.

In order to visualize the deviation pattern from the covariance plane through gait cycle, the distance from each point to the covariance plane was measured and a Kernel method (Kim and Scott, 2012) was used to create a heat map. This plot was performed over the covariance plane within the three-dimensional space of thigh, shank, and foot EA.

To evaluate the changes in the movement range of the EAs during gait performance, peak comparisons for each EA were performed. Gait cycles were extracted from the original data according to the movement of the toe and heel markers. To normalize the data, the time variable was discarded and the gait progression was accounted from 0 to 100% for each subject. The averaged cycle profile was obtained for each EA of each limb and comparisons were made from the max peaks, min peaks, and max-min difference. The obtained data was plotted for each patient’ individual limbs and split in EA before and after HAL intervention. Data from all healthy volunteers was averaged and plotted along as reference. Peak comparisons also were performed as described above for joint angles. Cycles were extracted from the original kinematic data for hip, knee, and ankle joints.

Statistical analysis was done by using a Wilcoxon signed rank test (paired for side comparisons and before-after HAL intervention; unpaired for patients’ comparisons against volunteers) analyzing the evolution of patients before and after HAL intervention or either condition against healthy volunteers. Due to sample size, a power test was applied after each analysis (10,000 times replication). Significance was considered when a *P*-value <0.05 was accompanied by an observed power (OP) >50% (Hoenig and Heisey, 2001). All statistical analysis was carried out by using custom made scripts on MATLAB [version 8.4.0.150421 (R2014B)] and RStudio (version 1.0.136).

## Results

Walking performance analysis showed an improvement in cadence (mean; pre: 52.54 ± 25.5 steps/min, post: 70.48 ± 16.6 steps/min, *P*-value: 0.04, OP: 25%) and a significant improvement in 6MWD (mean; pre: 97.93 ± 66.1 m, post: 217 ± 77.9 m, *P*-value: <0.01, OP: 74.5%), stride length (mean; pre: 0.29 ± 0.1 m, post: 0.42 ± 0.08 m, *P*-value: 0.015, OP: 59.4%) and walking speed (**Figure 1A**; mean; pre: 16.45 ± 10.1 m/min, post: 31.4 ± 13.2 m/min, *P*-value: <0.01, OP: 51.2%). Functional scores also showed a tendency of recovery for FMA (mean; pre: 20.6 ± 5.4, post: 25.5 ± 4.2, *P*-value: <0.01, OP: 35.4%), and significant improvement for FAC (mean; pre: 1.90 ± 0.7, post: 3.7 ± 0.6, *P*-value: <0.01, OP: 98%), FIM (mean; pre: 88.7 ± 13.4, post: 109.3 ± 10.7, *P*-value: <0.01, OP: 77.6%), and FIM locomotion (mean; pre: 55.5 ± 12.6, post: 75.2 ± 11.1, *P*-value: <0.01, OP: 73.4%) after HAL intervention (**Figure 1B**).

Kinematics analysis was used to understand changes in coordination given by alteration in the planar covariation of thigh, shank, and foot limb segments (**Figure 2A**, elevation angles). Healthy volunteers displayed a tear drop shaped pattern as previously described (**Figure 2A**, volunteer). In contrast, the loops corresponding to the patients’ analysis were distorted in both paretic and non-paretic limbs (**Figure 2B**). Additionally, the distribution size of the data over the plane was importantly reduced for patients before HAL intervention evidencing impaired intersegmental coordination (**Figure 2B**, before HAL intervention). An example from one patient (S7, **Table 1**) showed progressive loop recovery at the days of the first, fourth, and seventh HAL interventions where enlargement of the loop width is evident and the paretic side tendency to recover the tear drop shape of the loop was noticeable (**Figure 2B**, day 18, 25, and 35 after stroke). After complete HAL intervention, recovery of the loop width for both sides (paretic and non-paretic) was found, with recovery of tear drop like pattern more accentuated for the paretic side (**Figure 2B**, after HAL intervention).

Comparisons of PC2 standard deviation (PC2-SD) were significantly different from paretic and non-paretic limbs before and after HAL intervention when compared to healthy group. However, there was a tendency to recover for both sides after HAL intervention [**Figure 2C**, PC2-SD comparison and **Supplementary Tables S1**, **S2**. PC2-SD *P*-values; Par-pre Vs healthy volunteers (healthy): <0.01, OP: 96.1%; par-post Vs healthy: <0.01, OP: 96.3%; np-pre Vs healthy: <0.01, OP: 99.5%; np-post Vs healthy: <0.01, OP: 89.4%]. The PV as well showed a tendency of recovery for paretic and non-paretic limbs of patients after HAL intervention (**Figure 2C**, PV2 comparison; PV2 *P*-values; par-pre Vs par-post: 0.013, OP: 26.4%). These results suggest that HAL intervention induced a recovery trend by improving the actual and proportional width of the gait loop on the covariance plane.

Deviation from the covariance plane evaluated by PC3-SD comparisons showed improvement mainly for the np-post (**Figure 2C**, PC3-SD comparison and **Supplementary Tables S1**, **S2**. *P*-values; np-post Vs par-post: 0.019, OP: 57.1%; par-pre Vs healthy: 0.04, OP: 53.8%; par-post Vs healthy: <0.01, OP: 81.7%). Proportional deviation from covariation plane evaluated by PV3 demonstrated a tendency of recovery from patients after HAL intervention for non-paretic side as well (**Figure 2C**, PV3 comparison and PV3 *P*-values; par-pre Vs healthy: 0.03, OP: 53%). For paretic side, tendency of recovery was found when comparing between paretic and non-paretic limbs before and after HAL intervention (PV3 Par-pre Vs par-post *P*-value: 0.02, OP: 26.6%) and only paretic limbs before the intervention were significantly different from healthy volunteers. After HAL intervention, the PV3 reached values similar to healthy volunteers without exhibiting a statistical difference (**Figure 2C**; PV3 comparison *P*-values; np-pre Vs healthy: <0.01, OP: 92.2%; np-post Vs healthy: <0.01, OP: 74.6%). This tendency of recovery of PV3 suggests positive changes in planarity of coordination after HAL intervention. Other comparisons did not show significant differences.

Heat maps were used to graphically assess the deviation pattern from the covariance plane. The pattern found in healthy volunteers corresponded to a loop with two main hot spots in the areas related to heel strike and toe off (**Figure 2D**). Patients showed distorted patterns which differed importantly from the healthy pattern, encompassing a smaller surface, and evidencing one small hot spot (**Figure 2D**, before HAL). However, after HAL therapy, the loop shape was recovered for both sides (paretic and non-paretic), and the loop shape was closer to the volunteer conditions (**Figure 2D**, after HAL). The resemblance of heat maps after HAL intervention to healthy suggests an improvement in limb motion and plane stability.

**FIGURE 3:**
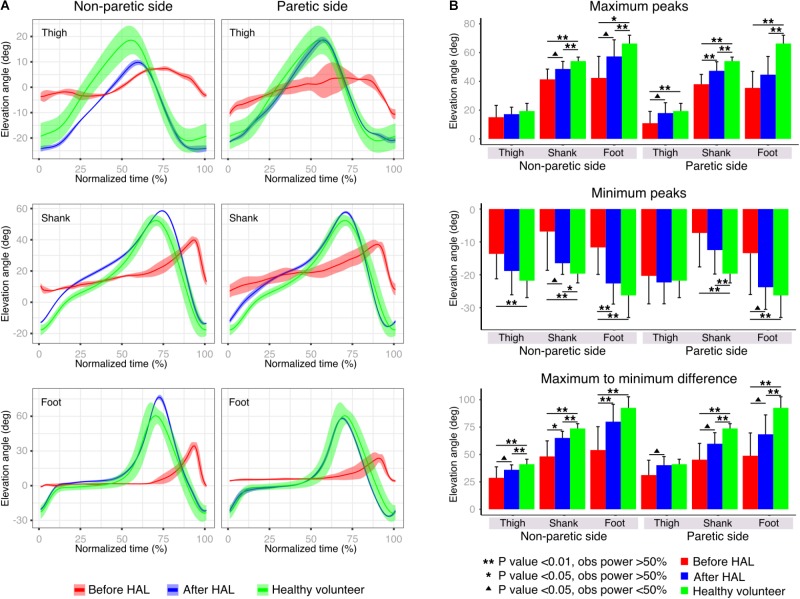
Peak analysis. **(A)** Elevation angle profiles were plotted before and after HAL therapy for each limb segment of one example patient’s paretic and non-paretic leg. The solid line represents the profile mean and width of highlighted area represents the standard deviation. Healthy volunteer plot (green line) is given by the averaged results of all healthy volunteer participants. **(B)** Maximum peaks, minimum peaks, and maximum to minimum peaks differences were compared for paretic and non-paretic side before and after HAL. As control, results from healthy volunteers were also included.

Peak comparisons before and after HAL intervention were used to evaluate the range of movement of each EA. Patients before HAL intervention showed lower peaks with an increased variability when compared to plots after HAL intervention; where the SD was smaller and the pattern resembled the healthy group data (**Figure 3A**). Statistical comparisons of max peaks, min peaks, and max-min difference were also performed (**Figure 3B** and **Supplementary Tables S3**, **S4**). Max peaks for non-paretic side did not show any significance related to thigh EA. Shank and foot showed significant difference from healthy volunteers before and after HAL intervention, but a tendency of recovery was noticed (shank *P*-value; np-pre Vs np-post: 0.04, OP: 44%; np-pre Vs healthy: <0.01, OP: 99.1%; np-post Vs healthy: <0.01, OP: 76.6%. Foot *P*-value; np-pre Vs np-post: 0.019, OP: 42.4%; np-pre Vs healthy: < 0.01, OP: 98.1%; np-post Vs healthy: 0.04, power test: 56.4%). On the paretic side, significant differences between thigh, shank, and foot before HAL intervention and healthy group were found; (thigh *P*-value; par-pre Vs healthy: <0.01, OP: 73.2%. Shank *P*-value: par-pre Vs healthy: <0.01, OP: 99.9%; par-post Vs healthy: <0.01, OP: 81.5%. Foot *P*-value; par-pre Vs healthy: <0.01, power test 99.9%; par-post Vs healthy: <0.01, power test: 99%). Peak comparisons before and after HAL intervention showed significant difference only for shank (*P*-value: par-pre Vs par-post: <0.01, OP: 60.7%) but tendency of recovery was evident for all EA. Min peaks showed a tendency of recovery more marked for the non-paretic side, where thigh EA peaks showed a significant difference before HAL intervention and healthy but not after HAL intervention and healthy (thigh *P*-value; np-pre Vs healthy: <0.01, OP: 75.9%; np-post Vs healthy: 0.22). Shank min peaks showed statistical difference before and after HAL intervention against healthy, but tendency of recovery also was found (shank *P*-value; np-pre Vs np-post: <0.01, OP: 41.5%; np-pre Vs healthy: <0.01, OP: 84.4%; np-post Vs healthy: <0.02, OP: 59.63%). Foot min peaks showed a significant difference before and after HAL intervention, and also, significant difference was found before HAL intervention and healthy only (foot *P*-value; np-pre Vs np-post: <0.01, OP: 66.04%; np-pre Vs healthy: <0.01, power test 98.2%; np-post Vs healthy: 0.22). On the paretic limb, there were no relevant changes for thigh min peaks. On the other hand, shank and foot min peaks showed a tendency of recovery being more marked for foot peaks where significant difference was found only before HAL intervention and healthy volunteers (shank *P*-value; par-pre Vs healthy: <0.01, OP: 88.6%; par-post Vs healthy: <0.01, OP: 76.0%. Foot *P*-value; par-pre Vs healthy: <0.01, power test 75.4%; par-post Vs healthy: 0.43). Finally, the max-min difference showed a tendency of recovery mainly marked for non-paretic foot where a significant difference was found before and after HAL intervention and between before HAL intervention and healthy group only (thigh *P*-value; np-pre Vs healthy: <0.01, OP: 89.2%; np-post Vs healthy: <0.01, OP: 69.2%. Shank *P*-value; np-pre Vs np-post: 0.019, OP: 68.3%; np-pre Vs healthy: <0.01, OP: 99.4%; np-post Vs healthy: <0.01, OP: 92.4%. Foot *P*-value; np-pre Vs np-post: <0.01, OP: 58.4%; np-pre Vs healthy: <0.01, power test 99.5%; np-post Vs healthy: 0.051). The paretic side also showed a tendency of improvement before and after HAL intervention. Results from shank and foot evidenced significance when comparing peaks before and after HAL to healthy volunteers. However, peaks obtained after HAL intervention tended to increase to the level of the healthy group (shank *P*-value; par-pre Vs par-post: <0.01, OP: 43.7%; par-pre Vs healthy: <0.01, OP: 99.7%; par-post Vs healthy: <0.01, OP: 94.2%. Foot *P*-value; par-pre Vs par-post: <0.01, OP: 35.4%; par-pre Vs healthy: <0.01, OP: 99.9%; par-post Vs healthy: <0.01, OP: 92.8%). Peak analysis results showed important changes related principally to the foot EA for non-paretic side and thigh EA for paretic side. Despite the maintained significance between patients after HAL intervention and healthy volunteers, and overall tendency of recovery was also found for shank EA. These changes may represent an improvement in toe clearance and limb excursion induced by HAL intervention.

In addition to EA analysis, peaks comparison was also performed for hip, knee, and ankle joint angles (**Supplementary Figure S1**). Before HAL, patients showed relatively flat plots. However, after HAL intervention, resemblance to healthy volunteers improved (**Supplementary Figure S1A**). Max peaks comparison showed a tendency of recovery for knee angle of the non-paretic side (**Supplementary Figure S1B** and **Supplementary Tables S5**, **S6**. Knee *P*-value: np-pre Vs healthy: <0.01, OP: 97.9%; np-post Vs healthy: 0.017, OP: 65.8%) although pre-post comparisons did not show significance. Min peaks comparisons did not find significance related to improvement. Max to min peaks comparison of non-paretic side showed marginal significance before and after HAL intervention for knee joint (*P*-value np-pre Vs np-post: 0.03, OP: 33.3%) and significant difference when comparing patients before and after HAL intervention against healthy volunteers (*P*-value np-pre Vs healthy: <0.01, OP: 99.8%; np-post Vs healthy: <0.01, OP: 93.4%). However, a tendency of recovery was noted. Comparisons for the paretic side evidenced significance related to improvement for hip joint angle, where comparisons before and after HAL intervention showed a marginal improvement (*P*-value: < 0.01, OP: 42.1). Additionally, comparisons before HAL intervention and healthy were statistically different, but when comparing healthy volunteers to patients after HAL intervention, significance was not found (*P*-value par-pre Vs healthy: <0.01, OP: 85.3; par-post Vs healthy: 0.29). This data reinforces the findings related to thigh and shank EA improvement after HAL intervention. Lack of significance related to other joint peaks may be related to the high variability found on joint angles analysis between subjects and between trials in healthy volunteers (Borghese et al., 1996). Due to gait deterioration in stroke patients, gait cycle variability inter-trial and inter-subject is expected to be larger. Given their stereotyped characteristics (Borghese et al., 1996; Ivanenko et al., 2008), EA analysis allows collection of less variable data which is desirable in populations with gait disturbances as stroke patients.

## Discussion

Gait generation is a complex dynamic process with spinal circuits able to generate basic locomotion patterns, altered by descending pathways carrying information from the brain. The system also has feedback from muscle receptors, skin afferents, and senses which help to gather information to adapt the locomotion pattern to the environment (Belda-Lois et al., 2011). However, the disruption of the descending pathways after the ischemia onset induce muscle co-activation of the paretic limb (Beyaert et al., 2015) instead of selective control of individual joint movements adapting to the new conditions in order to stabilize gait (Verma et al., 2012). In hemiparesis, the underlying mechanisms of asymmetry are poor single limb support and lack of forward movement control (Verma et al., 2012) summed to the disproportion between hemilateral motor commands leading to bilateral coordination deficit (Meijer et al., 2011). This asymmetry leads to compensation strategies leaning on the capabilities of the non-paretic limb. The achieved walking pattern is characterized by a higher proportion of the gait cycle spent in the unaffected single limb support and bipedal support in comparison to the supporting time provided by the hemiparetic leg (Olney and Richards, 1996), drifting away from regaining a coordinated gait pattern.

It has been acknowledged that planar covariation of limb segments may be a way used by the central nervous system to reduce the effective degrees of freedom thus simplifying the control of posture and locomotion (Lacquaniti et al., 1990, 2002; Ivanenko et al., 2008). During gait, the rotation of EA belonging to thigh, shank, and foot limb segments covary tracing a tridimensional trajectory of temporal changes lying close to a plane in healthy subjects (Borghese et al., 1996; Ivanenko et al., 2008). The planar law of intersegmental coordination is discussed to be representing the strategy of modularized control of the lower limbs by the central nervous system in terms of limb orientation and length (Ivanenko et al., 2007, 2008). Since healthy subjects’ trajectories correspondence to plane has minimal variation, plane fitting related data may not be so relevant; however, distorted gait patterns have an effect over this aspect. Previous studies using planar covariation analysis in different pathologies affecting locomotion as Parkinson (Grasso et al., 1999), stroke (Bleyenheuft et al., 2009; MacLellan et al., 2013; Chow and Stokic, 2015), myelopathy (Puentes et al., 2018), and cerebellar ataxia (Martino et al., 2014) have demonstrated the preservation of intersegmental coordination to some extent despite the underlying condition; however, impaired intersegmental coordination was found in all cases. Interestingly, an additional research documenting changes in planar covariation of amputees found preservation of the gait loop and adequate intersegmental coordination on novice and experienced prostheses users (Leurs et al., 2012). The integrity of the central nervous system and the preserved intersegmental coordination in such patients suggest that the relationship between segments may have a central control and that it is not dependent on biomechanical constrains (Ivanenko et al., 2008; Leurs et al., 2012). The improvement of planar covariation, may suggest changes related to neural plasticity leading to coordination and gait recovery (Poppele and Bosco, 2003; Ivanenko et al., 2008).

In the present study, the initial evaluation before HAL intervention confirmed the conservation of the law of intersegmental coordination. However, the hemiparetic gait induced distortion and size reduction of the gait loop showing impaired intersegmental coordination for both paretic and non-paretic limbs (**Figure 2B**). Early HAL intervention was able to induce recovery of the covariation loop shape and distance to plane distribution (**Figures 2B,D**). PCA comparisons also showed a tendency of recovery for the actual width of the loop and plane stability more accentuated for the non-paretic limb (PC2-SD, **Figure 2C**). On the other hand, the proportion of variance of loop width and proportional plane stability recovery was more prominent for the paretic side (PV3, **Figure 2C**). The tendency of recovery shown by the planar covariation analysis suggests a beneficial effect of HAL intervention on improvement of gait coordination.

Previous studies have shown lack of recovery of spatiotemporal coordination in gait of stroke patients after conventional rehabilitation despite improvement of balance ability and walking velocity (Patterson et al., 2008, 2014). Foot drag during swing phase, foot slap, unstable support, and weak propulsion during stance phase are the reasons which lead the non-paretic limb to adopt compensatory movements to compromise gait. The compensatory movements deviate the non-paretic limb from its original movement pattern, even to deteriorate lateral asymmetry of gait (Olney and Richards, 1996). We hypothesize that HAL intervention was able to modify gait coordination by providing functional control to the paretic limb at the earliest possible occasion post stroke. HAL allowed to perform voluntary control of the paretic leg by its function to provide joint motion assistance in accordance with the patients’ bioelectrical activity. This feature enabled patients to perform larger foot excursion during swing phase, smoother landing, better support stability, and empowered propulsion during stance phase. This characteristic may have saved the non-paretic limb from the demand to take compensatory movements through recovery, probably contributing to bilateral improvement of gait coordination. By using the patient’s own bioelectric signals, the gait output can be perceived natural and coherent, generating sensory feedback of the performed motion over to the central nervous system. A recent report by our group (Tan et al., 2018) analyzed the muscle synergies of some of the patients included in our research before and after HAL intervention. It was found that before HAL intervention, stroke patients had a reduced number of muscle synergies of the paretic leg when comparing to the contralateral limb. HAL intervention improved in some cases the synergies of the paretic side but in some cases also a reduction of synergies in the non-paretic side was found. This synergies reduction of the non-paretic side may be a strategy to balance bilateral muscle control to improve gait coordination. In contrast to conventional rehabilitation strategies, where the goal is to adapt compensatory movements to enhance performance in daily life activities (Verma et al., 2012), HAL provided support allowing a more natural recovery process thus improving coordination.

Peak analysis of the EA showed a trend of recovery more accentuated for thigh peaks in the paretic limb; on the other hand, shank and foot peak recovery seemed to be more favored for the non-paretic limb (**Figure 3B**). We think HAL intervention improved the range of movement of lower limbs during gait through improvement of hip flexion which is known to be affected in hemiparetic gait (Olney and Richards, 1996). Also, we think that HAL support to the hemiparetic side allowed a better performance of the non-paretic leg, balancing the gait and allowing a normal time for swing and stance phases of both limbs reducing the early foot contact of the non-paretic limb evidenced in stroke patients (Olney and Richards, 1996). Joint angle analysis showed tendency of improvement of knee angle in the non-paretic side and also hip angle of the paretic side (**Supplementary Figure S1B**), detecting less significant comparisons than the EA analysis. We consider that the main difference in the results is related to the high variability of joint angles regarding gait pattern and velocity in contrast to the stereotyped pattern found on EA (Borghese et al., 1996).

It is known that exercising is an effective non-invasive treatment able to induce neuroplasticity in the central nervous system and increase resistance to the brain damage (Cotman and Berchtold, 2002; Sandrow-Feinberg and Houlé, 2015). Offering rehabilitation in the early stages of stroke increases the functional recovery and reduces hospitalization time, favoring the patient to continue rehabilitation in the outpatient clinic. However, delayed admission to rehabilitation program is common for stroke patients leading to poor outcomes and long-term disability (Salter et al., 2006). We believe that HAL intervention may have a higher impact in an early stage avoiding the establishment of compensatory strategies which affect importantly gait coordination in this population. Additionally, studies analyzing adaptability changes in the post-stroke brain have found evident contralesional activation in the early stages which returns to normal in the subacute and chronic stages of the disease (Beyaert et al., 2015). This activation is supposed to correspond to automatic and intentional cognitive processes for support, balance, and progression that help patients to find strategies to generate gait despite hemiparesis (Beyaert et al., 2015). We hypothesize that patients receiving early HAL intervention may use this mechanism to find better strategies to improve motor output reducing abnormal postures and allowing a better gait recovery process.

This study has limitations with respect to the size of the population included for analysis. Also, we have not included a control group of stroke patients undergoing conventional rehabilitation to compare with patients after HAL intervention. Additionally, changes in the non-paretic side and its improvement after HAL intervention lead us to the question whether hemiparetic patients may respond differently to HAL if the double leg version is used to support also the non-paretic side. Further studies shall enlarge the population size, adding a control group of patients receiving standard rehabilitation only and comparing the output in stroke patients after an early intervention by using the single leg and double leg HAL exoskeleton.

## Author Contributions

SP and HK collected, analyzed, and interpreted the data, and wrote and drafted the manuscript. HW administered HAL therapy and collected the data for clinical scores. TU and MY contributed in the development of HAL intervention program. YS originally developed the robot suit HAL and conceived the idea of HAL intervention. AM diagnosed the patients and prescribed HAL intervention, also provided important comments for the clinical part of the study, and contributed in the design of HAL intervention protocol. KS designed the analysis and provided essential insight for the paper. All authors made critical revisions of the manuscript and approved the final version. All authors read and approved the final manuscript.

## Conflict of Interest Statement

YS is the CEO, shareholder, and director of CYBERDYNE Inc. which manufacture the robot suit HAL. CYBERDYNE was not involved in the study design, data collection, analysis, writing or submission of this article. The remaining authors declare that the research was conducted in the absence of any commercial or financial relationships that could be construed as potential conflict of interest.

## Abbreviations

6MWD: 6-Minute Walk Distance
EA: elevation angles
FAC: Functional Ambulation Classification
FIM: Functional Independence Measure
FMA: Fugl-Meyer Assessment
HAL: Hybrid Assistive Limb
max peaks: maximum peaks
min peaks: minimum peaks
max-min difference: maximum peak to minimum peak difference
np-pre: non-paretic side before HAL intervention
np-post: non-paretic side after HAL intervention
par-post: paretic side after HAL intervention
par-pre: paretic side before HAL intervention
PC2SD: PCA second component standard deviation
PC3SD: PCA third component standard deviation
PCA: Principal Component Analysis
PV2: PCA second component percentage of variance
PV3: PCA third component percentage of variance
