# Supplementary material for: Reshaping of Bilateral Gait Coordination in Hemiparetic Stroke Patients After Early Robotic Intervention
Source: Front Neurosci. 2018 Oct 9;12:719. doi: 10.3389/fnins.2018.00719 (PMC6189332; doi:10.3389/fnins.2018.00719)
Supplement: Supplementary file 1 [file Data_Sheet_1.pdf]

Supplementary Material

Reshaping of bilateral gait coordination in hemiparetic stroke patients after early robotic intervention.

Sandra Puentes*[1,2], Hideki Kadone[2], Hiroki Watanabe[3], Tomoyuki Ueno[4], Masashi Yamazaki[5], Yoshiyuki Sankai[3], Aiki Marushima[6], Kenji Suzuki[3].

*** Correspondence: Sandra Puentes: sandra@ccr.tsukuba.ac.jp**

Limb	Data	Mean before HAL	Mean after HAL
**Paretic**	PC2-SD	8.35 ± 3.4	9.56 ± 2.5
**Non-paretic**	PC2-SD	8.29 ± 2.7	10.6 ± 2.1
**Healthy**	PC2-SD	13.3 ± 1.1	-
**Paretic**	PV2	0.16 ± 0.05	0.12 ± 0.05
**Non-paretic**	PV2	0.17 ± 0.09	0.13 ± 0.04
**Healthy**	PV2	0.14 ± 0.02	-
**Paretic**	PC3-SD	2.62 ± 1.1	2.61 ± 0.6
**Non-paretic**	PC3-SD	2.82 ± 1.1	3.58 ± 0.76
**Healthy**	PC3-SD	3.44 ± 0.5	-
**Paretic**	PV3	0.02 ± 0.01	0.009 ±0.004
**Non-paretic**	PV3	0.02 ± 0.008	0.01 ± 0.005
**Healthy**	PV3	0.009 ± 0.002	-

Supplementary Table 1. PCA averaged data for patients and volunteers. Healthy volunteers' data was taken in a single session and analyzed regardless limb side (Healthy). Data from patients was taken before the first and after the last HAL intervention and analyzed depending on the functional status of the limb (Paretic, Non-paretic).

Data	Comparison	P-value	Observed power (%)
**PC2-SD**	Par-pre Vs Par-post	0.193	8.3%
**PC2-SD**	Np-pre Vs Np-post	<0.01	32.8%
**PC2-SD**	Np-pre Vs Par-pre	0.846	4.0%
**PC2-SD**	Np-post Vs Par-post	0.461	10.5%
**PC2-SD**	Par-pre Vs Healthy	**<0.01**	**96.1%**
**PC2-SD**	Par-post Vs Healthy	**<0.01**	**96.4%**
**PC2-SD**	Np-pre Vs Healthy	**<0.01**	**99.5%**
**PC2-SD**	Np-post Vs Healthy	**<0.01**	**89.4%**
**PV2**	Par-pre Vs Par-post	0.014	26.5%
**PV2**	Np-pre Vs Np-post	0.160	16.3%
**PV2**	Np-pre Vs Par-pre	0.846	4.7%
**PV2**	Np-post Vs Par-post	0.846	4.8%
**PV2**	Par-pre Vs Healthy	0.191	32.3%
**PV2**	Par-post Vs Healthy	0.245	19.2%
**PV2**	Np-pre Vs Healthy	0.356	29.5%
**PV2**	Np-post Vs Healthy	0.464	10.5%
**PC3-SD**	Par-pre Vs Par-post	0.770	4.1%
**PC3-SD**	Np-pre Vs Np-post	0.064	23.1%
**PC3-SD**	Np-pre Vs Par-pre	0.922	5.1%
**PC3-SD**	Np-post Vs Par-post	**0.020**	**57.2%**
**PC3-SD**	Par-pre Vs Healthy	**0.045**	**53.8%**
**PC3-SD**	Par-post Vs Healthy	**<0.01**	**81.7%**
**PC3-SD**	Np-pre Vs Healthy	0.072	34.0%
**PC3-SD**	Np-post Vs Healthy	0.555	8.4%
**PV3**	Par-pre Vs Par-post	0.027	26.7%
**PV3**	Np-pre Vs Np-post	0.084	20.0%
**PV3**	Np-pre Vs Par-pre	0.770	4.0%
**PV3**	Np-post Vs Par-post	0.020	42.8%
**PV3**	Par-pre Vs Healthy	**0.035**	**53.0%**
**PV3**	Par-post Vs Healthy	0.621	8.1%
**PV3**	Np-pre Vs Healthy	**<0.01**	**92.2%**
**PV3**	Np-post Vs Healthy	**<0.01**	**74.7%**

Supplementary Table 2. Statistical comparisons were performed by using a Wilcoxon signed rank test. The analysis was paired for side comparisons and pre-post HAL intervention comparisons, and unpaired for patients' comparisons against healthy volunteers. Following, a post-hoc power test (10.000 repetitions) was used and statistical significance was considered when a P-value <0.05 was accompanied by an observed power >50% (Par: paretic, Np: non-paretic, pre: before HAL, post: after HAL, Healthy: healthy volunteers).

Data	Mean max peaks (deg)	Mean min peaks (deg)	Mean max-min diff (deg)
**Np-pre (thigh)**	15.06 ± 8.2	-13.64 ± 7.5	28.71 ± 10
**Np-pre (shank)**	41.38 ± 7.1	-6.84 ± 11.8	48.23 ± 14.2
**Np-pre (foot)**	42.38 ± 15.01	-11.67 ± 8.2	54.05 ± 21.3
**Np-post (thigh)**	17.18 ± 4.8	-18.84 ± 7.2	36.03 ± 4.52
**Np-post (shank)**	48.62 ± 5.2	-16.48 ± 3.4	65.11 ± 6.1
**Np-post (foot)**	57.31 ± 11.5	-22.63 ± 6.2	79.94 ± 15.8
**Par-pre (thigh)**	10.89 ± 8.2	-20.33 ± 8.5	31.22 ± 13.5
**Par-pre (shank)**	37.99 ± 6.86	-7.28 ± 10.3	45.27 ± 14.8
**Par-pre (foot)**	35.44 ± 11.4	-13.42 ± 12.5	48.87 ± 20.7
**Par-post (thigh)**	17.94 ± 7.2	-22.32 ± 6.5	40.27 ± 7.8
**Par-post (shank)**	47.31 ± 6.3	-12.48 ± 7.3	59.8 ± 10.1
**Par-post (foot)**	44.64 ± 12.6	-23.78 ± 6.7	68.43 ± 17.6
**Healthy (thigh)**	19.35 ± 5.3	-21.80 ± 5.1	41.16 ± 4.5
**Healthy (shank)**	54.08 ± 2.7	-19.65 ± 2.8	73.74 ± 4.4
**Healthy (foot)**	66.30 ± 5.7	-26.26 ± 6.7	92.56 ± 10.1

Supplementary Table 3. Averaged data from peak analysis of thigh, shank and foot elevation angles before and after HAL intervention for patients depending on the functional status of the limb (paretic, non-paretic), and single data collection for healthy volunteers regardless limb side. (Par: paretic, Np: non-paretic, pre: before HAL, post: after HAL, Healthy: healthy volunteers).

Comparison	Peak	P-value	Observed power (%)
**Np-pre Vs Np-post**	Max thigh	0.557	6.7%
**Np-pre Vs Np-post**	Max shank	0.049	44.0%
**Np-pre Vs Np-post**	Max foot	0.020	42.4%
**Np-pre Vs Healthy**	Max thigh	0.146	27.7%
**Np-pre Vs Healthy**	Max shank	**<0.01**	**99.1%**
**Np-pre Vs Healthy**	Max foot	**<0.01**	**98.2%**
**Np-post Vs Healthy**	Max thigh	0.265	14.5%
**Np-post Vs Healthy**	Max shank	**<0.01**	**76.7%**
**Np-post Vs Healthy**	Max foot	**0.045**	**56.5%**
**Np-pre Vs Np-post**	Min thigh	0.275	18.7%
**Np-pre Vs Np-post**	Min shank	<0.01	41.5%
**Np-pre Vs Np-post**	Min foot	**<0.01**	**66.0%**
**Np-pre Vs Healthy**	Min thigh	**<0.01**	**75.9%**
**Np-pre Vs Healthy**	Min shank	**<0.01**	**84.4%**
**Np-pre Vs Healthy**	Min foot	**<0.01**	**98.3%**

**Np-post Vs Healthy**	Min thigh	0.226	19.3%
**Np-post Vs Healthy**	Min shank	**0.027**	**59.6%**
**Np-post Vs Healthy**	Min foot	0.226	21.6%
**Np-pre Vs Np-post**	Diff thigh	0.027	31.8%
**Np-pre Vs Np-post**	Diff shank	**0.020**	**68.3%**
**Np-pre Vs Np-post**	Diff foot	**<0.01**	**58.5%**
**Np-pre Vs Healthy**	Diff thigh	**<0.01**	**89.3%**
**Np-pre Vs Healthy**	Diff shank	**<0.01**	**99.4%**
**Np-pre Vs Healthy**	Diff foot	**<0.01**	**99.5%**
**Np-post Vs Healthy**	Diff thigh	**<0.01**	**69.3%**
**Np-post Vs Healthy**	Diff shank	**<0.01**	**92.4%**
**Np-post Vs Healthy**	Diff foot	0.051	53.1%
**Par-pre Vs Par-post**	Max thigh	0.010	29.7%
**Par-pre Vs Par-post**	Max shank	**<0.01**	**60.8%**
**Par-pre Vs Par-post**	Max foot	0.064	22.0%
**Par-pre Vs Healthy**	Max thigh	**<0.01**	**73.3%**
**Par-pre Vs Healthy**	Max shank	**<0.01**	**99.9%**
**Par-pre Vs Healthy**	Max foot	**<0.01**	**99.9%**
**Par-post Vs Healthy**	Max thigh	0.796	8.9%
**Par-post Vs Healthy**	Max shank	**<0.01**	**81.5%**
**Par-post Vs Healthy**	Max foot	**<0.01**	**99.0%**
**Par-pre Vs Par-post**	Min thigh	0.557	5.7%
**Par-pre Vs Par-post**	Min shank	0.131	14.1%
**Par-pre Vs Par-post**	Min foot	0.010	36.3%
**Par-pre Vs Healthy**	Min thigh	0.408	9.7%
**Par-pre Vs Healthy**	Min shank	**<0.01**	**88.7%**
**Par-pre Vs Healthy**	Min foot	**0.010**	**75.4%**
**Par-post Vs Healthy**	Min thigh	0.944	6.3%
**Par-post Vs Healthy**	Min shank	**<0.01**	**76.0%**
**Par-post Vs Healthy**	Min foot	0.436	12.1%
**Par-pre Vs Par-post**	Diff thigh	0.014	24.3%
**Par-pre Vs Par-post**	Diff shank	<0.01	43.8%
**Par-pre Vs Par-post**	Diff foot	<0.01	35.4%
**Par-pre Vs Healthy**	Diff thigh	0.121	55.8%
**Par-pre Vs Healthy**	Diff shank	**<0.01**	**99.8%**
**Par-pre Vs Healthy**	Diff foot	**<0.01**	**99.9%**
**Par-post Vs Healthy**	Diff thigh	0.760	9.2%
**Par-post Vs Healthy**	Diff shank	**<0.01**	**94.3%**
**Par-post Vs Healthy**	Diff foot	**<0.01**	**92.9%**

Supplementary Table 4. Statistical comparisons from peaks analysis of elevation angles were performed by using a Wilcoxon signed rank test. The analysis was paired for side comparisons and pre-post HAL intervention comparisons, and unpaired for patients' comparisons against healthy volunteers. Following, a post-hoc power test (10.000 repetitions) was used and statistical significance was considered when a P-value <0.05 was accompanied by an observed power >50% (Par: paretic, Np: non-paretic, pre: before HAL, post: after HAL, Healthy: healthy volunteers).

Data	Mean max peaks (deg)	Mean min peaks (deg)	Mean max-min diff (deg)
**Np-pre (hip)**	39.52 ± 11.2	-1.26 ± 9.83	40.78 ± 9.6
**Np-pre (knee)**	20.62 ± 10.6	-12.39 ± 16.2	33.01 ± 11.2
**Np-pre (ankle)**	18.69 ± 6	-6.28 ± 7.2	24.9 ± 9.6
**Np-post (hip)**	48.08 ± 10.6	-1.13 ± 8.3	49.21 ± 7.54
**Np-post (knee)**	27.92 ± 9.1	-11.53 ± 8.8	39.45 ± 3.9
**Np-post (ankle)**	19.55 ± 6.8	-10.75 ± 7.8	30.30 ± 6.8
**Par-pre (hip)**	37.83 ± 12.8	2.62 ± 11.1	35.21 ± 14.6
**Par-pre (knee)**	23.04 ± 9.7	-4.82 ± 15.7	27.86 ± 11.7
**Par-pre (ankle)**	17.28 ± 7.1	-2.05 ± 5.6	19.33 ± 4.7
**Par-post (hip)**	36.59 ± 16.1	0.41 ± 9.2	36.18 ± 13.2
**Par-post (knee)**	28.18 ± 7.11	-10.67 ± 10.1	38.85 ± 7.4
**Par-post (ankle)**	18.31 ± 9.06	-1.63 ± 8.3	19.95 ± 6.61
**Healthy (hip)**	57.87 ± 6.3	-1.98 ± 6.5	59.85 ± 4.8
**Healthy (knee)**	23.89 ± 6.3	-17.16 ± 8.1	41.05 ± 3.3
**Healthy (ankle)**	18.67 ± 6.7	-10.93 ± 6.4	29.6 ± 6.1

Supplementary Table 5. Averaged data from peak analysis of hip, knee and ankle joint angles before and after HAL intervention. Data from patients was taken before the first and after the last HAL intervention and analyzed depending on the functional status of the limb (paretic, non-paretic). Healthy volunteers' data was taken in a single session and analyzed regardless limb side (Healthy: healthy volunteers, Par: paretic, Np: non-paretic, pre: before HAL, post: after HAL).

Comparison	Peak	P-value	Observed power (%)
**Np-pre Vs Np-post**	max hip	0.359	20.5%
**Np-pre Vs Np-post**	max knee	0.164	22.2%
**Np-pre Vs Np-post**	max ankle	0.734	4.6%
**Np-pre Vs Healthy**	max hip	0.463	15.5%
**Np-pre Vs Healthy**	max knee	**<0.01**	**97.9%**
**Np-pre Vs Healthy**	max ankle	0.900	3.9%
**Np-post Vs Healthy**	max hip	0.375	21.6%
**Np-post Vs Healthy**	max knee	**0.017**	**65.8%**
**Np-post Vs Healthy**	max ankle	0.705	5.2%
**Np-pre Vs Np-post**	min hip	1.000	3.9%
**Np-pre Vs Np-post**	min knee	1.000	4.2%
**Np-pre Vs Np-post**	min ankle	0.164	14.5%
**Np-pre Vs Healthy**	min hip	0.900	16.8%
**Np-pre Vs Healthy**	min knee	0.860	7.3%
**Np-pre Vs Healthy**	min ankle	0.176	30.2%
**Np-post Vs Healthy**	min hip	0.298	30.4%
**Np-post Vs Healthy**	min knee	0.821	6.6%
**Np-post Vs Healthy**	min ankle	0.596	6.0%
**Np-pre Vs Np-post**	diff hip	0.164	21.2%
**Np-pre Vs Np-post**	diff knee	0.039	33.4%
**Np-pre Vs Np-post**	diff ankle	0.496	16.1%
**Np-pre Vs Healthy**	diff hip	0.106	5.6%
**Np-pre Vs Healthy**	diff knee	**<0.01**	**99.8%**
**Np-pre Vs Healthy**	diff ankle	0.145	26.7%
**Np-post Vs Healthy**	diff hip	0.403	16.3%
**Np-post Vs Healthy**	diff knee	**<0.01**	**93.4%**
**Np-post Vs Healthy**	diff ankle	0.561	6.1%
**Par-pre Vs Par-post**	max hip	0.426	14.4%
**Par-pre Vs Par-post**	max knee	0.820	4.1%
**Par-pre Vs Par-post**	max ankle	0.820	4.6%
**Par-pre Vs Healthy**	max hip	0.940	7.8%
**Par-pre Vs Healthy**	max knee	**<0.01**	**97.4%**
**Par-pre Vs Healthy**	max ankle	0.528	6.8%
**Par-post Vs Healthy**	max hip	0.253	28.5%
**Par-post Vs Healthy**	max knee	**<0.01**	**92.9%**
**Par-post Vs Healthy**	max ankle	0.860	6.3%

**Par-pre Vs Par-post**	min hip	0.301	9.2%
**Par-pre Vs Par-post**	min knee	0.570	5.2%
**Par-pre Vs Par-post**	min ankle	0.910	4.1%
**Par-pre Vs Healthy**	min hip	0.053	56.6%
**Par-pre Vs Healthy**	min knee	0.298	13.1%
**Par-pre Vs Healthy**	min ankle	**<0.01**	**88.9%**
**Par-post Vs Healthy**	min hip	0.194	34.8%
**Par-post Vs Healthy**	min knee	0.528	11.3%
**Par-post Vs Healthy**	min ankle	**<0.01**	**75.7%**
**Par-pre Vs Par-post**	diff hip	**<0.01**	**42.2%**
**Par-pre Vs Par-post**	diff knee	0.820	4.4%
**Par-pre Vs Par-post**	diff ankle	0.910	4.1%
**Par-pre Vs Healthy**	diff hip	**<0.01**	**85.3%**
**Par-pre Vs Healthy**	diff knee	**<0.01**	**98.7%**
**Par-pre Vs Healthy**	diff ankle	**<0.01**	**98.4%**
**Par-post Vs Healthy**	diff hip	0.298	1.7%
**Par-post Vs Healthy**	diff knee	**<0.01**	**99.4%**
**Par-post Vs Healthy**	diff ankle	**<0.01**	**90.7%**

Supplementary Table 6. Statistical comparisons from peaks analysis of joint angles were performed by using a Wilcoxon signed rank test. The analysis was paired for side comparisons and pre-post HAL intervention comparisons, and unpaired for patients' comparisons against healthy volunteers. Following, a post-hoc power test (10.000 repetitions) was used and statistical significance was considered when a P-value <0.05 was accompanied by an observed power >50% (Par: paretic, Np: non-paretic, pre: before HAL, post: after HAL, Healthy: healthy volunteers).

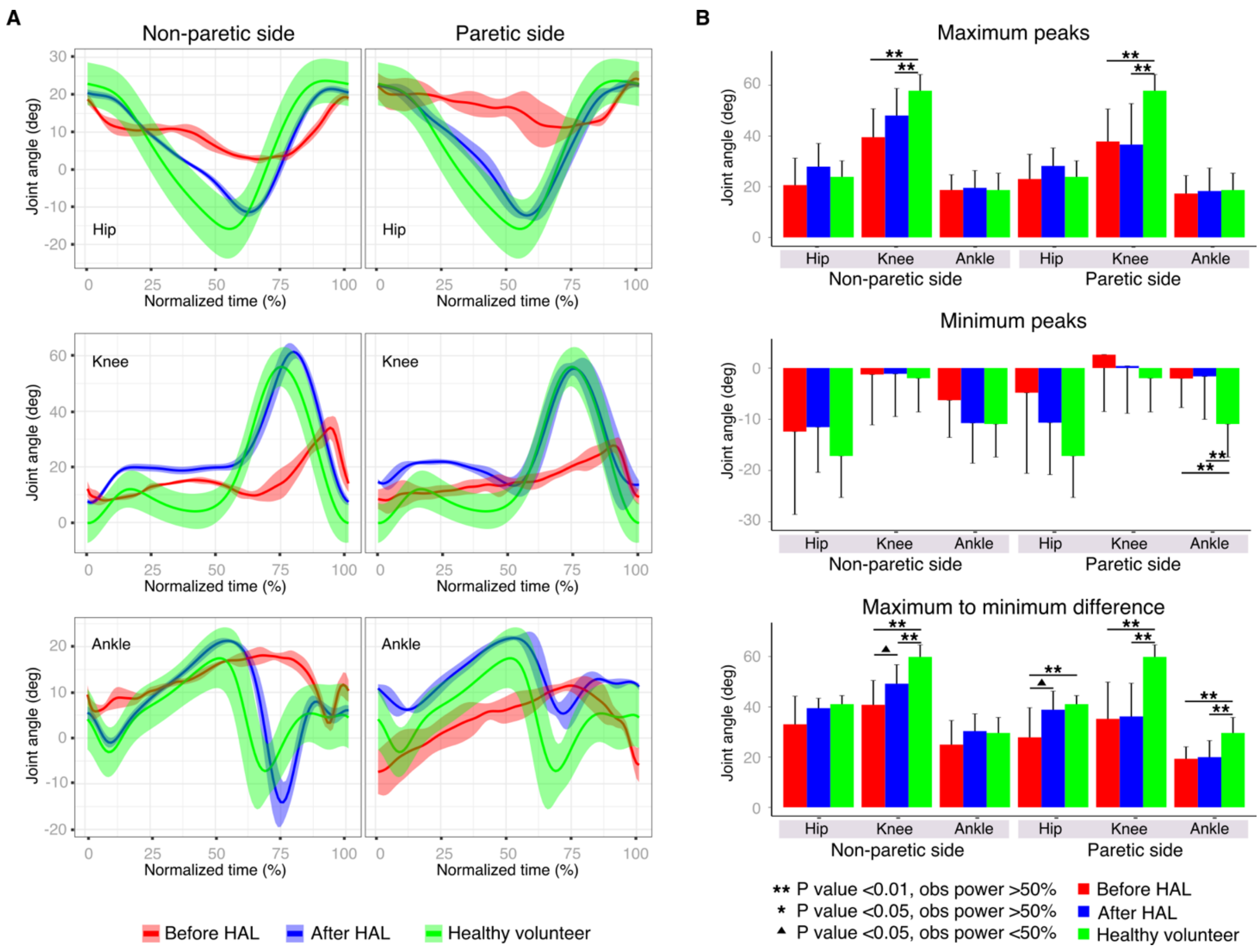

Figure S1. Peak analysis. (A) Joint angle profiles were plotted before and after HAL therapy for each limb segment of one example patient's paretic and non-paretic leg. The solid line represents the profile mean and width of highlighted area represents the standard deviation. Healthy volunteer plot (green line) is given by the averaged results of all healthy volunteer participants. (B) Maximum peaks, minimum peaks and maximum to minimum peaks differences were compared for paretic and non-paretic side before and after HAL. As control, results from healthy volunteers were also included.
